# Life Course Modifiable Risk Factors for Dementia Stratified by ApoE-4 Status

**DOI:** 10.1093/geroni/igaf122.2174

**Published:** 2025-12-31

**Authors:** Victoria Williams, Ralph Trane, Kamil Sicinski, Carol Roan, Pamela Herd, Michal Engelman, Sanjay Asthana

**Affiliations:** University of Wisconsin–Madison School of Medicine and Public Health, Madison, Wisconsin, United States; University of Wisconsin–Madison School of Medicine and Public Health, Madison, Wisconsin, United States; University of Wisconsin–Madison, Madison, Wisconsin, United States; University of Wisconsin–Madison, Madison, Wisconsin, United States; University of Michigan, Ann Arbor, Michigan, United States; University of Wisconsin-Madison, Madison, Wisconsin, United States; University of Wisconsin–Madison School of Medicine and Public Health, Madison, Wisconsin, United States

## Abstract

The 2024 Lancet Commission identified 14 modifiable risk factors reliably associated with increased risk for dementia. However, dementia is a complex condition influenced by both genetic predisposition and environmental factors, with gene by environment interactions often overlooked in risk models. The apolipoprotein (ApoE-4) allele confers the strongest genetic risk for Alzheimer’s disease, while also shown to modify the effects of environmental exposures on the dementia phenotype. We applied the Lancet risk factor model to a single cohort of 5,526 participants in the Wisconsin Longitudinal Study (WLS). Risk factors were defined by Lancet cut-points and life-course timing criteria, drawing from 70 years of prospectively collected WLS data. We used logistic regression with multiple imputation to model dementia outcomes by the Lancet model risk factors (also controlling for age and sex), stratified by ApoE-4 carrier status. Replication of the 2024 Lancet model in a single cohort revealed only a restricted subset of modifiable risk factors that associated with late-life dementia status. Among APoE-4 carriers only, we found that mid-life diabetes increased dementia odds, whereas mid-life hypertension reduced risk for dementia. Among ApoE-4 non-carriers, only mid-life hearing loss and diabetes associated with increased dementia odds. Overall, we observed slightly divergent dementia risk profiles based on ApoE-4 status, such that mid-life diabetes emerged as the primary risk factor for dementia among ApoE-4 carriers, whereas mid-life hearing loss was found to be most strongly associated with dementia risk among ApoE-4 non-carriers. Resultant findings highlight the importance of considering gene-by-environment interactions when formulating dementia risk models.

